# Correction: Møller, P.; et al. Causes for Frequent Pathogenic *BRCA1* Variants Include Low Penetrance in Fertile Ages, Recurrent De-Novo Mutations and Genetic Drift. *Cancers* 2019, *11*, 132

**DOI:** 10.3390/cancers12020410

**Published:** 2020-02-10

**Authors:** Pål Møller, Mev Dominguez-Valentin, Einar Andreas Rødland, Eivind Hovig

**Affiliations:** 1 Department of Tumor Biology, Institute of Cancer Research, The Norwegian Radium Hospital, Part of Oslo University Hospital, 0424 Oslo, Norway; mev.dominguez.valentin@rr-research.no (M.D.-V.); einarro@ulrik.uio.no (E.A.R.); ehovig@ifi.uio.no (E.H.); 2 Center for Hereditary Tumors, HELIOS-Klinikum Wuppertal, University ofWitten-Herdecke, 42283 Wuppertal, Germany; 3 Department of Medical Genetics, The Norwegian Radium Hospital, Oslo University Hospital, 0424 Oslo, Norway; 4 Center for Bioinformatics, Department of Informatics, University of Oslo, PO box 1080, Blindern, 0316 Oslo, Norway

The authors wish to make the following corrections to this paper [1]:

The authors would like to replace Table 3 in [1]. The corrections are correcting typographical errors when translating our database in BIC format to HGVS nomenclature, and removing four carriers which had zero follow-up time. The original version of Table 3 is:

and should be replaced with the following Table 3:

The authors would like to apologize for any inconvenience caused to the readers by these changes.

## Figures and Tables

**Table 3:** *Path_BRCA1* variants detected.

*BRCA1* Variant	Number of Carriers
c.1016dupA	366
c.1556delA	337
c.3228_3229delAG	193
c.697_698delGT	159
c.3178G>T	119
c.4745delA	63
c.1A>C	49
c.2351_2357delCGTTACT	47
c.5075-2A>C	37
c.3084_3094delTAATAACATTA	36
c.5047G>T	35
c.(441 + 1_442–1)_(4357 + 1_4358–1)del	24
c.(4185 + 1_41861)_(4357 + 1_4358–1)dup	22
c.3607C>T	22
c.3048_3052dupTGAGA	21
c.5266dupC	20
c.3331_3334delCAAG	20
c.(5332 + 1_5333–1)_(5406 + 1_5407–1)del	19
c.1072delC	18
c.5511G>A	15
c.1450G>T	14
c.(80 + 1_81–1)_(4986 + 1_4987–1)del	12
c.5513T>G	12
c.66dupA	12
c.2475delC	11
c.3966delA	11
c.2869C>T	10
c.2591C>G	10
c.1058G>A	10
c.3319G>T	10
c.4065_4068delTCAA	9
c.5407-25T>A	8
c.1292dupT	7
c.2558ins356	7
c.3874delT	6
c.68_69delAG	6
c.5251C>T	6
c.5534C>A	5
c.1687C>T	5
c.(?_1–1)_(4357 + 1_4358–1)del	5
c.794_795delCT	5
c.5503C>T	5
c.3710delT	5
c.4035delA	4
c.2989_2990dupAA	4
c.4689C>G	4
c.3319G>T	4
c.5213G>A	4
c.4300_4301delA	4
c.115T>G	4
c.5153G>C	3
c.848T>A	3
c.339_361dup	3
c.4612C>T	3
c.(?_1–1)_(134 + 1_135–1)del	3
c.457_458ins21	3
c.3770_3771delAG	3
c.2869C>T	3
c.3700_3704delGTAAA	2
c.3477_3479delAAAinsC	2
c.3835_3835delG	2
c.2438dupG	2
c.2389G>T	2
c.1695dupG	2
c.65T>C	2
c.1287_1287delA	2
c.385_385delG	2
c.2681_2682delAA	2
c.4932_4933dupAA	2
c.4972_4972delA	2
c.(5074 + 1_5075–1)_(5592 + 1_?-1)del	2
c.(134 + 1_135–1)_(441 + 1_442–1)del	2
c.241C>T	1
c.5434C>G	1
c.3817C>T	1
c.2722G>T	1
(4185 + 1_41861)_(4357 + 1_4358–1)del	1
c.4883T>C	1
c.1059G>A	1
c.5123C>T	1
c.3937C>T	1
c.140G>T	1
c.1961dupA	1
c.3808T>G	1
c.(5193 + 1_5194–1)_(5592 + 1_?-1)del	1
c.514C>T	1
c.2185G>T	1
c.4689C>G	1
c.130T>A	1
c.4987-16T>G	1
c.1674_1674dupA	1
c.2745_2748delTCAA	1
c.5075A>C	1
c.(4675 + 1_4676–1)_(4986 + 1_4987–1)del	1
c.929delA	1
c.3005delA	1
c.5212G>A	1
Sum	1918

**Table 3:** *Path_BRCA1* variants detected.

*BRCA1* Variant	Number of Carriers
c.1016dupA	366
c.1556delA	337
c.3228_3229delAG	193
c.697_698delGT	159
c.3178G>T	119
c.4745delA	63
c.1A>G	49
c.2351_2357delCGTTACT	47
c.5075-2A>C	37
c.3084_3094delTAATAACATTA	36
c.5047G>T	35
del exon 8–13	24
c.3607C>T	22
dup exon 13	22
c.3048_3052dupTGAGA	21
c.3331_3334delCAAG	20
c.5266dupC	20
del exon 22	19
c.1072delC	18
c.5511G>A	15
c.1450G>T	14
c.3319G>T	14
c.2869C>T	13
c.5513T>G	12
c.66dupA	12
del exon 3-16	12
c.2475delC	11
c.3966delA	11
c.1058G>A	10
c.2591C>G	10
c.4065_4068delTCAA	9
c.5407-25T>A	8
c.1292dupT	7
c.2558ins356	7
c.3874delT	6
c.457_458ins21	6
c.5251C>T	6
c.68_69delAG	6
c.1687C>T	5
c.3710delT	5
c.5503C>T	5
c.5534delA	5
c.794_795delCT	5
del exon 1–13	5
c.4689C>G	5
c.115T>G	4
c.2989_2990dupAA	4
c.4035delA	4
c.4300delA	4
c.5213G>A	4
c.3770_3771delAG	3
c.4612C>T	3
c.5153G>C	3
c.848T>A	3
del exon 1–3	3
c.1287delA	2
c.1695dupG	2
c.2389G>T	2
c.2438dupG	2
c.2681_2682delAA	2
c.3477_3479delAAAinsC	2
c.3700_3704delGTAAA	2
c.3835delG	2
c.386delG	2
c.4932_4933dupAA	2
c.4972delA	2
c.65T>C	2
del exon 18–24	2
del exon 5–7	2
c.1059G>A	1
c.130T>A	1
c.140G>T	1
c.1674dupA	1
c.1961dupA	1
c.2185G>T	1
c.241C>T	1
c.2722G>T	1
c.2727_2730delTCAA	1
c.3005delA	1
c.3817C>T	1
c.3937C>T	1
c.5075A>C	1
c.514C>T	1
c.5212G>A	1
c.5434C>G	1
c.929delA	1
del exon 13	1
del exon 16	1
del exon 20–24	1
Sum	1914
