# Supplementary material for: Causes for Frequent Pathogenic BRCA1 Variants Include Low Penetrance in Fertile Ages, Recurrent De-Novo Mutations and Genetic Drift
Source: Cancers (Basel). 2019 Jan 23;11(2):132. doi: 10.3390/cancers11020132 (PMC6406718; doi:10.3390/cancers11020132)
Supplement: Supplementary file 1 [file cancers-11-00132-s001.pdf]

Methods for estimating and comparing incidence rates that vary with subjects' age

1 Introduction

This document summarises methods used in a series of published articles to estimate and compare cumulative incidence rates of cancer by age. However, none of them give a complete overview, or in-depth explanations.

The methods, although used to estimate cumulative cancer incidence rates, it should be applicable to other types of diseases or events.

A common approach to compute cumulative incidence rates is to use Kaplan-Meyer curves. However, when the time factor is the age of the subjects, the number of subjects at risk at the lower and upper ends of the age range may be low. This will cause incidence rates at these age periods to be uncertain. This will in turn affect the estimates for cumulative incidence rates.

For example, if a study includes subjects age 20 and above and follows them for a time period of up to 10 years, there will be very few subjects at risk at 20 years of age. Recall that a subject included at the age of 20 will be at risk from age 20 to age 30 if followed for 10 years, and the only subjects at risk at age 20 will be those included at age 20: a subject included at age 21 will not contribute to the number of subjects at risk at age 20. Consequently, estimates of risk at age 20 will have large uncertainties. To the contrary, subjects at risk at age 25 will include all subjects included before the age of 25, except those who leave the study either due to an event or from dropping out before the 10 year study period has passed, and is therefore more likely to comprise sufficient number of subjects to allow accurate risk estimates.

The method described in this document attempts to alleviate this problem by assuming that incidence rates vary slowly with age, and may be treated as fairly constant within age categories, eg using 5 year age intervals.

2 Methods

The data material consists of a number of subjects, each followed for a known time period, either until an event occurs or until they leave the study for other reasons. The number of events and time at risk are then aggregated per age interval.

The definitions and formulas are based on viewing each year under risk as the observational unit.

2.1 Incidence rates and cumulative incidence

If, in a given age interval, there are X events and a total of Y years at risk, the **annual incidence rate (*AIR*)** is $AIR = X/Yrs$. Estimates are made for each age interval, and referred to as $AIR(age)$ for each corresponding age.

The **cumulative incidence (*Q*)** for a given age is given by $Q(age) = Q(age-1) + [1 - Q(age-1)] \cdot AIR(age)$, where we set $Q(age) = 0$ for $age < Age0$ where $Age0$ is the lowest age of inclusion. Note that this requires that there are sufficient years of risk in the earliest age interval, otherwise the estimation errors in this age interval will affect all subsequent estimates. The cumulative incidence $Q(age)$ represents the portion of subjects who at that age has not experienced an event, assuming they satisfy the inclusion at age $Age0$ and have not subsequently been excluded for another reason.

In order to analyse estimation errors, we use the **hazard rate (*H*)**, defined as $H(age) = -\ln[1 - AIR(age)]$. The corresponding **cumulative hazard (*CH*)** is then defined as $CH(age) = CH(age-1) + H(age)$, where $CH(age) = 0$ for ages below the lowest age of inclusion: ie $CH(age) = H(Age0) + \cdots + H(age)$. The cumulative hazard corresponds to the cumulative incidence through the relation $CH(age) = -\ln[1 - Q(age)]$, or, conversely, $Q(age) = 1 - \exp[-CH(age)]$. Hazards are more convenient to analyse as they are defined as sums. Results derived on cumulative hazards may then be applied to cumulative incidences.

2.2 Error estimates

Uncertainties of the above point estimates are computed and presented as standard errors.

The **standard error of the annual incidence rate (*SEAIR*)** corresponding to an estimated *AIR* is estimated as $SEAIR = \sqrt{AIR \cdot (1 - AIR)/Y}$. This is denoted $SEAIR(age)$ for each age in the corresponding age interval.

The **standard error of the hazard rate (*SEH*)** is defined as $SEH(age) = SEAIR(age)/[1 - AIR(age)]$. This definition follows the result that if $V = f(U)$ for estimators U and V related by a function f, then their variation is related by $\delta V \approx f'(U) \cdot \delta U$ so that $se(V) \approx f'(U) \cdot se(V)$.

The **standard error of the cumulative hazard (*SECH*)** may be found by adding variance estimates for the corresponding age intervals and taking the square root to convert the variance to standard error. This assumes that statistical errors in different age intervals are independent. Since the statistical errors of $H(age)$ are the same across each age interval, ie not independent, these must be treated differently: for a period of N years within a single age interval, the standard error is $N \cdot SEH$. Accordingly, the standard error of the cumulative hazard satisfies

$$SECH(age)^2 = \left(SEH(Age0) + \cdots\right)^2 + \cdots + \left(+ \cdots + SEH(age)\right)^2$$

where ages within each age interval are grouped together. Another way to write this is as

$$SECH(age)^2 = SECH(A-1)^2 + \left(SEH(A)^2 + \cdots + SEH(age)\right)^2 = SECH(A-1)^2 + \left(N \cdot SEH(age)\right)^2$$

where A is the first age within the age interval, and $N = age - A + 1$ is the number of years at risk within this age interval.

The **standard error of the cumulative incidence (SEQ)** is defined as $SEQ(age) = SECH(age) \cdot [1 - Q(age)]$, following the same relation as between *SEH* and *SEAIR*.

2.3 Confidence intervals and comparisons

Let $1 - \alpha$ be the confidence level, eg 0.05 for 95% confidence levels, and let $z = z_{\alpha/2}$ be the upper $\alpha/2$ percentile of the standard normal distribution. Ie, for $G_N(z) = \Pr[N(0,1) > z]$ the upper tail probabilities of the standard normal distribution, $G(z_u) = u$.

We assume, as an approximation, that the estimators *AIR* and *Q* are normally distributed. This seems to be an ok assumption when the number of events is sufficient to give a fairly accurate estimate. Potential problems will be discussed later.

Assuming that *Q* is normally distributed with standard error *SEQ*, gives **confidence intervals of the cumulative incidence** $Q \pm z \cdot SEQ$: ie the $(1-\alpha) \times 100\%$ confidence interval of $Q(age)$ is $[Q(age) - z \cdot SEQ(age), Q(age) + z \cdot SEQ(age)]$. Note that the normal distribution assumption means that negative values are initially permitted, which does not make sense: the natural thing is then to truncate the interval at zero, and conclude that the method may be somewhat inaccurate as the actual distribution is likely to deviate considerably from the normal distribution.

We compare cumulative incidence of two groups, A and B, by testing the hypothesis $Q_A = Q_B$. Since we assume that the estimators are independent and approximately normally distributed, the difference $Q_A - Q_B$ is normally distributed with standard error $SEQ_{A-B} = \sqrt{SEQ_A^2 + SEQ_B^2}$: ie $Z_{A-B} = (Q_A - Q_b)/SEQ_{A-B}$ is standard normally distributed, and statistically significant at the α level if $|Z_{A-B}| > z_{\alpha/2}$. The P-value of the two-sided hypothesis test is then $P = 2 \cdot G_N(|Z_{A-B}|)$.

Let $R = Q_A/Q_B$ be the **cumulative incidence ratio** between groups A and B. In order to determine the coinfidence interval of *R*, we assess $Q_{A-rB} = Q_A - r \cdot Q_B$: testing the hypothesis $R = r$ is equivalent to testing $Q_{A-rB} = 0$. Once more assuming normal distribution, Q_{A-rB} has standard error $SEQ_{A-rB} = \sqrt{SEQ_A^2 + r^2 \cdot SEQ_B^2}$. The hypothesis $R = r$ may then be excluded if $|Q_{A-rB}| > z \cdot SEQ_{A-rB}$. The **confidence interval of the cumulative incidence ratio** thus consists of all $r \geq 0$ for which $|Q_{A-rB}| \leq z \cdot SEQ_{A-rB}$. The bounds of the confidence interval, ie where the inequality becomes an equality, may be found by solving the equation

$$Q_{A-rB}^2 = z^2 \cdot SEQ_{A-rB}^2 \iff Q_A - r \cdot Q_B = SEQ_A^2 + r^2 \cdot SEQ_B^2$$

keeping in mind that negative solutions may arise in cases where $R = 0$ or $R = \infty$ are not excluded: while in theory these should not be possible, the normal distribution assumption on Q allows confidence intervals to pass into negative values, which of course do not make sense.

3 Discussion

There are a few modelling choices for which alternative choices might have been taken.

The choice to treat the observation year as the observational unit, ie the definition of $Q(age)$, had been made prior to the development of the remaining methods. Alternatively, computations could have been made starting with the hazard rates which treats time in a more continous fashion. The main difference would have been to replace the standard error estimate $SEAIR = AIR \cdot (1 - AIR)/Yrs$, which is based on a binomial distribution, and a definition of SEH based on this, with an alternative definition of SEH based on a Poisson distribution of the number of events, which could then be applied to $SEAIR$. However, the difference would for the most part be very minor so long as AIR is small (in comparison to one).

The assumption that Q has an approximate normal distribution, permits negative values, which is obviously inappropriate. This may happen if the number of events underlying the estimate is small. An alternative could have been to assume that some transformation of Q is normal. Assuming the $\ln Q$ is normally distributed instead eliminates the possibility of negative values, but tends to give confidence intervales that are too skewed, as found on simulated data.
